# Factors associated with achieving the 10-minute scene-to-ECG transmission target in prehospital care: the role of EMS agency-level cumulative ECG transmissions

**DOI:** 10.1186/s12873-026-01630-8

**Published:** 2026-05-29

**Authors:** Hiroki Sato, Kunio Yufu, Tatsunori Nakashima, Hidefumi Akioka, Ryuzo Abe, Tsuyoshi Shimomura, Naohiko Takahashi

**Affiliations:** 1 Disaster Management Center, Oita University Hospital, Idaigaoka 1-1, Yufu, Yufu, Oita 879-5593 Japan; 2 Department of Cardiology and Clinical Examination, Faculty of Medicine, Oita University, Yufu, Japan; 3 Department of Cardiology, Oita Red Cross Hospital, Oita, Japan; 4 Department of Emergency Medicine, Faculty of Medicine, Oita University, Yufu, Japan

**Keywords:** Acute coronary syndrome, Electrocardiogram, Emergency medical services, Prehospital care

## Abstract

**Background:**

Prehospital 12-lead electrocardiogram (ECG) improves outcomes in ST-segment elevation myocardial infarction, yet meeting the 10-minute target from first medical contact remains difficult. We evaluated determinants of faster prehospital ECG performance.

**Methods:**

We analysed a prefecture-wide registry in Japan (September 2021 to August 2025) of patients with suspected acute coronary syndrome who had prehospital ECG transmission. Prehospital ECG performance was assessed using the scene-to-ECG interval, from emergency medical services (EMS) on-scene arrival to ECG transmission. The primary outcome was scene-to-ECG ≤ 10 min; the secondary outcome was interval length. Agency experience was measured by cumulative ECG transmissions. Mixed-effects logistic and linear models with agency random intercepts were used with adjustment for age, sex, and time of arrival.

**Results:**

Of 1,748 patients, 672 (38.4%) achieved ≤ 10 min; the median scene-to-ECG was 12 min (interquartile range 9 to 17). Greater cumulative transmissions were associated with higher odds of ≤ 10 min (adjusted odds ratio 1.07 per 100 cases, 95% confidence interval 1.02 to 1.14) and shorter intervals (β − 0.29 min per 100 cases, 95% confidence interval − 0.39 to − 0.19). Older age, female sex, and nighttime arrival were associated with lower odds of achieving ≤ 10 min, whereas older age and nighttime arrival were associated with longer intervals.

**Conclusions:**

At the EMS agency level, higher cumulative ECG transmissions were associated with achieving the ≤ 10-minute scene-to-ECG target. This suggests that focused onboarding and continued EMS training may enhance prehospital ECG performance. In the main analysis, older age, female sex, and nighttime arrival were associated with lower odds of achieving the target.

**Supplementary Information:**

The online version contains supplementary material available at 10.1186/s12873-026-01630-8.

## Background

Prehospital acquisition of a 12-lead electrocardiogram (ECG), often transmitted for remote interpretation, in patients with suspected acute coronary syndrome (ACS) enables early prenotification and activation of the cardiac catheterisation laboratory. This process facilitates destination triage to percutaneous coronary intervention (PCI)–capable hospitals and direct transfer to the catheterisation laboratory, thereby reducing door-to-device time and improving outcomes in patients with ST-segment elevation myocardial infarction (STEMI) [1–3]. Early ECG acquisition and transmission may also facilitate fibrinolytic reperfusion by shortening in-hospital door-to-needle time [4] and supporting prehospital fibrinolysis when indicated [5]. Although door-to-device time, defined as the interval from hospital arrival to first device activation, has been a central focus of STEMI care systems, further improvement requires optimization of the prehospital phase [6].

During this phase, rapid on-scene ECG acquisition at the first medical contact (FMC) by emergency medical services (EMS) is a key modifiable factor that shortens the FMC-to-device interval [7]. Current guidelines recommend that EMS personnel acquire and interpret a 12-lead ECG within 10 min of FMC in patients with suspected ACS, aligning with the 10-minute benchmark after emergency department (ED) arrival [5, 8, 9]. In ED settings, numerous studies have evaluated the door-to-ECG interval (ED arrival to first ECG). Across diverse populations, female sex, older age, and atypical presenting symptoms are consistently associated with longer door-to-ECG intervals [10–13], whereas standardised triage protocols, ED staff training, and workflow optimization reduce these delays [14–17].

In contrast, relatively few investigations have assessed the prehospital FMC-to-ECG interval among patients with suspected ACS or STEMI [7, 18–21]. Notably, prior studies indicate that more than half of patients did not receive an ECG within 10 min of FMC [7, 18, 19, 21]. Longer FMC-to-ECG intervals have been linked to female sex [18, 19, 21], older age [18], and absence of chest pain as the primary complaint [18]. However, determinants of prehospital ECG interval remain incompletely defined, and evidence regarding system-level interventions that reliably shorten this interval is limited. Therefore, this study aimed to identify factors associated with timely prehospital ECG transmission, measured as the FMC-to-ECG interval, among patients with suspected ACS who underwent completed ECG transmission, focusing on prehospital workflow processes relevant to early ECG-based triage.

## Methods

### Prehospital ECG transmission system in Oita Prefecture

Oita Prefecture, located on the northeastern coast of Kyushu in southwestern Japan, has a population of approximately 1.1 million. A mobile ECG transmission system (Cloud Cardiology; Labtech, Debrecen, Hungary) integrated with a secure cloud server (SCUNA; Mehergen, Fukuoka, Japan) operates as part of a cloud-based emergency support network [22]. EMS personnel acquire 12-lead ECGs on scene for patients with suspected ACS and upload them to the cloud server, which transmits the ECGs to hospitals for immediate prearrival interpretation. After transmission, the ECG is reviewed through the cloud system by a cardiologist at the hospital consulted for ECG interpretation. Based on the transmitted ECG and prehospital information, the cardiologist can provide feedback to EMS personnel regarding suspected ACS and the appropriate transport destination. When STEMI is suspected, the transmitted information can also support prearrival preparation for emergency catheterisation [23]. Details of ECG interpretation, hospital feedback, and subsequent instructions were not systematically recorded in the registry and were therefore unavailable for analysis. EMS personnel are instructed to consider prehospital 12-lead ECG acquisition when ACS is suspected during the initial on-scene assessment, particularly in patients with chest pain or chest discomfort [23]. However, prehospital ECG acquisition is not governed by a strict prefecture-wide checklist or a mandatory symptom-duration criterion, and the final decision is made by EMS personnel at the scene. As of 2025, the system was implemented in 24 hospitals and 57 of 64 ambulances in Oita Prefecture, representing a near-prefecture-wide emergency support network serving approximately 1.1 million residents. The 24 participating hospitals comprised 19 PCI-capable hospitals and five designated regional hub hospitals without PCI capability [22]. The 19 PCI-capable hospitals included all four emergency and critical care centres in the prefecture [22]. Emergency and critical care centres correspond to tertiary emergency medical institutions in Japan and provide advanced multidisciplinary emergency care for critically ill or severely injured patients. PCI-capable hospitals were defined as hospitals able to perform PCI, although not all of the 19 PCI-capable hospitals provided continuous 24-hour PCI availability. The five designated regional hub hospitals supported regional emergency care and ECG consultation within the network [22]. These facilities represented the major emergency and cardiovascular care institutions in the prefecture.

### Study design

This retrospective study analysed registry data from patients whose prehospital 12-lead ECGs were transmitted via the Oita prehospital ECG system between September 2021 (registry initiation) and August 2025. Eleven of 14 regional EMS agencies participated. For each case, agencies provided patient-level data on sex and age, along with prehospital timestamps for emergency call receipt, EMS on-scene arrival, ECG transmission, scene departure, and hospital arrival. The registry did not include structured information on the initial EMS alert reason, presenting symptoms, or specific reason for prehospital ECG acquisition. Hospital-phase information, including final diagnoses, treatments (e.g., whether PCI was performed), and outcomes, was not collected. The transmitted ECGs were not centrally reviewed for diagnostic classification in the present analysis.

The study complied with the Declaration of Helsinki and was approved by the Human Research Committee of Oita University (approval no. 3239). In accordance with the Ethical Guidelines for Medical and Health Research Involving Human Subjects established by the Japanese Ministry of Health, Labour, and Welfare, individual informed consent was not required. Study details were publicly disclosed, and participants were provided an opt-out option.

### Outcome

The scene-to-ECG interval was defined as the time from EMS on-scene arrival to completion of ECG transmission, calculated by subtracting the recorded arrival time from the system-logged transmission time. Because the registry records on-scene arrival but not the exact time of FMC, the scene-to-ECG interval was analysed instead of the FMC-to-ECG interval. This metric encompasses several on-scene steps, including patient assessment, identification of the need for a 12-lead ECG, electrode placement, acquisition, and transmission. Therefore, a shorter interval represents a more efficient diagnostic process. Because this measure begins at on-scene arrival rather than FMC, it imposes a stricter benchmark than guideline standards.

The primary outcome was achievement of a scene-to-ECG interval ≤ 10 min, consistent with recommendations for completing and interpreting a 12-lead ECG within 10 min. The secondary outcome was the scene-to-ECG interval as a continuous variable. Patients without completed ECG transmission were not included in the analytic cohort because the scene-to-ECG interval could not be defined. The registry recorded the time of completed transmission but did not systematically capture failed transmission attempts or reasons for transmission delay.

### Candidate factors for outcomes

Prespecified covariates potentially associated with the scene-to-ECG interval included three domains: (1) patient demographics (sex and age); (2) EMS on-scene arrival time, categorised as daytime (09:00–17:59) or nighttime (18:00–08:59, next day); and (3) the EMS agency–level cumulative number of ECG transmissions completed before the index patient’s transmission (i.e., the total number previously completed by that agency). The cumulative count served as a proxy for agency experience.

### Statistical analysis

Continuous variables were summarised as mean with standard deviation or median with interquartile range, and compared using the Student’s t test or Wilcoxon rank-sum test, as appropriate. Comparisons across more than two groups were conducted with the Kruskal–Wallis test. Categorical variables were expressed as counts and percentages and compared using the chi-square test. Scene-to-ECG intervals were compared by patient characteristics (sex and age), EMS on-scene arrival time (daytime vs. nighttime), and EMS agency.

The primary outcome was achievement of a scene-to-ECG interval ≤ 10 min. Generalised linear mixed-effects models with a logit link and a random intercept for EMS agency were used. The primary exposure was each agency’s cumulative number of prior prehospital 12-lead ECG transmissions. Three prespecified models with incremental adjustment were evaluated:

Model 1: cumulative number of prior prehospital 12-lead ECG transmissions.

Model 2: Model 1 plus sex and age.

Model 3: Model 2 plus on-scene arrival time (daytime vs. nighttime).

For descriptive analyses, the study period was divided into four consecutive 12-month intervals: September 1, 2021–August 31, 2022; September 1, 2022–August 31, 2023; September 1, 2023–August 31, 2024; and September 1, 2024–August 31, 2025.

The secondary outcome was the scene-to-ECG interval in minutes, analysed using linear mixed-effects models with identical covariate sets and a random intercept for EMS agency.

Missing data were handled using multiple imputation with fully conditional specification (100 imputations, fixed random seed). The imputation model included all analysis variables and selected prehospital timestamps as auxiliary variables. Estimates were combined using Rubin’s rules. Sensitivity analyses included complete-case analyses and reanalyses excluding patients with scene-to-ECG intervals above the 97.5th percentile of the overall distribution. We also repeated the mixed-effects models using an alternative categorisation of on-scene arrival time, with daytime defined as 07:00–21:59 and nighttime as 22:00–06:59. Statistical significance was defined as *p* < 0.05. All analyses were conducted using SAS software, version 9.4 (SAS Institute Inc., Cary, NC).

## Results

### Patient characteristics and EMS-related intervals

**Fig. 1:**
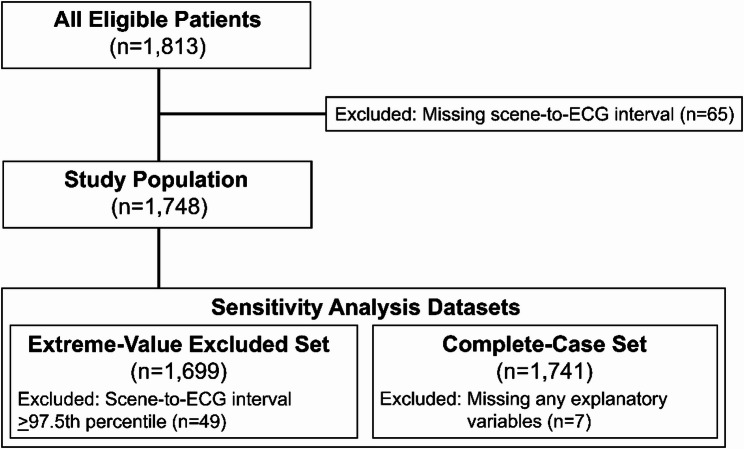
Flow diagram. Among eligible patients (n = 1,813), those with missing scene-to-ECG intervals were excluded (n = 65), resulting in a final study population of n = 1,748. For sensitivity analyses, two datasets were created: (1) an extreme-value–excluded dataset removing patients with scene-to-ECG intervals ≥ 97.5th percentile (≥ 34 min; n = 1,699) and (2) a complete-case dataset excluding patients missing any explanatory variable (n = 1,741). Abbreviations: ECG, electrocardiogram

A total of 1,748 patients were enrolled (Fig. 1). The median scene-to-ECG interval was 12 min (interquartile range [IQR], 9–17), with 672 patients (38.4%) achieving ≤ 10 min. Baseline characteristics by study period are summarised in Table 1. No significant differences were observed across the four annual periods in sex, age, on-scene arrival time, or prehospital time intervals; however, the distribution of cases across EMS agencies differed significantly.

**Table 1:** Baseline characteristics of patients with ECG transmission

Variables	Period 1	Period 2	Period 3	Period 4	Total	*p* value
	Sep2021 - Aug2022	Sep2022 - Aug2023	Sep2023 - Aug2024	Sep2024 - Aug2025		
	*n* = 378	*n* = 505	*n* = 485	*n* = 380	*n* = 1,748	
Sex, n (%), *n* = 1,743						
Male	227 (60.1)	330 (65.9)	284 (58.7)	244 (64.2)	1085 (62.2)	0.078
Female	151 (40.0)	171 (34.1)	200 (41.3)	136 (35.8)	658 (37.8)	
Age, year, mean (SD), *n* = 1,746	75.0 (14.0)	74.2 (14.5)	74.7 (14.8)	73.4 (15.4)	74.3 (14.7)	0.490
On-scene arrival time*, n (%), *n* = 1,748						
Daytime	213 (56.4)	275 (54.5)	249 (51.3)	192 (50.5)	929 (53.1)	0.310
Nighttime	165 (43.7)	230 (45.5)	236 (48.7)	188 (49.5)	819 (46.9)	
Prehospital time interval†, minutes, median [IQR]‡						
Call to scene, *n* = 1,747	8.0 [7.0–11.0]	8.0 [7.0–11.0]	8.0 [6.0–11.0]	8.0 [6.0–11.0]	8.0 [6.0–11.0]	0.588
Scene-to-ECG, *n* = 1,748	12.0 [9.0–17.0]	13.0 [9.0–18.0]	12.0 [9.0–17.0]	12.0 [9.0–16.0]	12.0 [9.0–17.0]	0.165
On-scene, *n* = 1,725	18.0 [13.0–23.0]	17.0 [13.0–23.0]	17.0 [12.0–23.0]	17.0 [13.0–23.0]	17.0 [13.0–23.0]	0.865
Scene-to-hospital, *n* = 1,717	12.0 [7.0–23.0]	13.0 [7.0–25.0]	13.0 [7.0–26.0]	15.0 [8.0–29.0]	13.0 [7.0–26.0]	0.084
EMS agency, n (%), *n* = 1,748						
#1	231 (61.1)	250 (49.5)	188 (38.8)	115 (30.3)	784 (44.9)	< 0.001
#2	0 (0.0)	3 (0.6)	8 (1.7)	19 (5.0)	30 (1.7)	
#3	1 (0.3)	4 (0.8)	9 (1.9)	6 (1.6)	20 (1.1)	
#4	72 (19.1)	93 (18.4)	87 (17.9)	95 (25.0)	347 (19.9)	
#5	13 (3.4)	6 (1.2)	8 (1.7)	10 (2.6)	37 (2.1)	
#6	25 (6.6)	44 (8.7)	28 (5.8)	26 (6.8)	123 (7.0)	
#7	4 (1.1)	2 (0.4)	0 (0.0)	0 (0.0)	6 (0.3)	
#8	4 (1.1)	32 (6.3)	31 (6.4)	20 (5.3)	87 (5.0)	
#9	10 (2.7)	14 (2.8)	16 (3.3)	13 (3.4)	53 (3.0)	
#10	14 (3.7)	22 (4.4)	49 (10.1)	32 (8.4)	117 (6.7)	
#11	4 (1.1)	35 (6.9)	61 (12.6)	44 (11.6)	144 (8.2)	

ECG indicates electrocardiogram; EMS, emergency medical services

* On-scene arrival time was categorised by the EMS on-scene arrival timestamp: Daytime, 09:00–17:59; Nighttime, 18:00–08:59 (spanning midnight to the following day)

† Call-to-scene: time from receipt of the emergency call to EMS on-scene arrival; Scene-to-ECG interval: time from EMS on-scene arrival to ECG transmission; On-scene time: time from EMS on-scene arrival to scene departure

‡ [IQR]: [25th percentile–75th percentile]

As shown in Table 2, patients with scene-to-ECG intervals ≤ 10 min were younger (mean ± SD, 72.9 ± 15.2 vs. 75.2 ± 14.3 years; *p* < 0.001) and less likely to be female (31.5% vs. 41.7%; *p* < 0.001) than those with intervals > 10 min. The distribution of EMS agencies also varied between these groups (*p* < 0.001).

**Table 2:** Baseline characteristics of patients by scene-to-ECG interval (*≤* 10 min vs. > 10 min)

Variables	Total	Scene-to-ECG	Scene-to-ECG	*P* value
		*≤* 10 min	> 10 min	
	*n* = 1748	*n* = 672	*n* = 1076	
Sex, n (%), *n* = 1,743				
Male	1085 (62.2)	459 (68.5)	626 (58.3)	< 0.001
Female	658 (37.8)	211 (31.5)	447 (41.7)	
Age, year, mean (SD), *n* = 1,746	74.3 (14.8)	72.9 (15.2)	75.2 (14.3)	< 0.001
On-scene arrival time*, n (%), *n* = 1,748				
Daytime	929 (53.1)	382 (56.9)	547 (50.8)	0.014
Nighttime	819 (46.9)	290 (43.2)	529 (49.2)	
Prehospital time interval†, minutes, median [IQR]‡				
Call to scene, *n* = 1,747	8.0 [6.0–11.0]	8.0 [7.0–11.0]	8.0 [6.0–11.0]	0.851
On-scene, *n* = 1,725	17.0 [13.0–23.0]	14.0 [11.0–19.0]	19.0 [14.0–25.0]	< 0.001
Scene-to-hospital, *n* = 1,717	13.0 [7.0–26.0]	10.0 [6.0–20.0]	16.0 [8.0–29.0]	< 0.001
EMS agency, n (%), *n* = 1,748				
#1	784 (44.9)	322 (47.9)	462 (42.9)	< 0.001
#2	30 (1.7)	9 (1.3)	21 (2.0)	
#3	20 (1.1)	4 (0.6)	16 (1.5)	
#4	347 (19.9)	182 (27.1)	165 (15.3)	
#5	37 (2.1)	10 (1.5)	27 (2.5)	
#6	123 (7.0)	25 (3.7)	98 (9.1)	
#7	6 (0.3)	2 (0.3)	4 (0.4)	
#8	87 (5.0)	14 (2.1)	73 (6.8)	
#9	53 (3.0)	31 (4.6)	22 (2.0)	
#10	117 (6.7)	19 (2.8)	98 (9.1)	
#11	144 (8.2)	54 (8.0)	90 (8.4)	

ECG indicates electrocardiogram; EMS, emergency medical services

* On-scene arrival time was categorised by the EMS on-scene arrival timestamp: Daytime, 09:00–17:59; Nighttime, 18:00–08:59 (spanning midnight to the following day)

† Call-to-scene: time from receipt of the emergency call to EMS on-scene arrival; Scene-to-ECG interval: time from EMS on-scene arrival to ECG transmission; On-scene time: time from EMS on-scene arrival to scene departure

‡ [IQR]: [25th percentile–75th percentile]

### Scene-to-ECG intervals

**Fig. 2:**
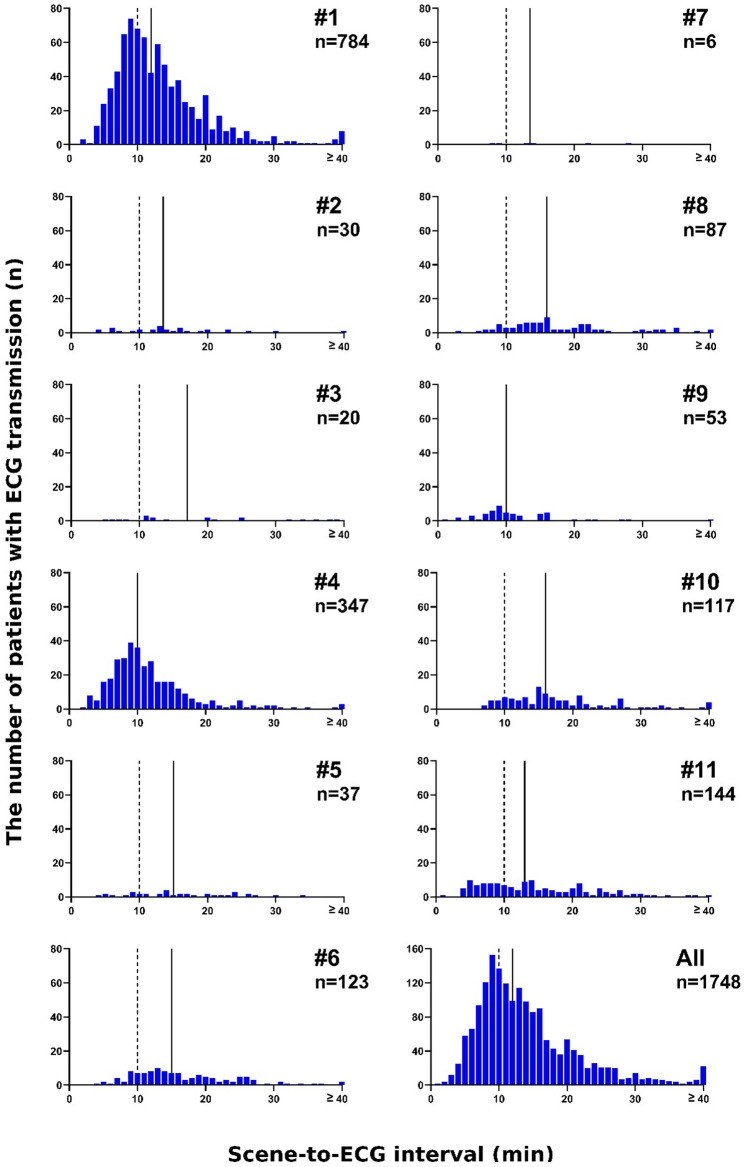
Distribution of the scene-to-ECG intervals across EMS agencies. Histograms depict the distribution of scene-to-ECG intervals (minutes) for each EMS agency (#1–#11) and for all patients combined. The y-axis represents the number of patients with ECG transmissions. Black vertical lines indicate the median scene-to-ECG interval for each agency. Abbreviations: ECG, electrocardiogram; EMS, emergency medical services

As summarised in Table 3, scene-to-ECG intervals were shorter in males (male vs. female: 12.0 [9.0–17.0] vs. 13.0 [10.0–17.0] minutes; *p* = 0.001) and in daytime arrivals (daytime vs. nighttime: 12.0 [9.0–16.0] vs. 13.0 [9.0–18.0] minutes; *p* = 0.026). The interval did not differ by age (< 75 vs. ≥ 75 years; *p* = 0.170) but varied significantly across EMS agencies (Kruskal–Wallis *p* < 0.001). Figure 2 illustrates agency-specific distributions, showing right-skewed patterns for agencies #1 and #4, which had higher cumulative numbers of ECG transmissions.

**Table 3:** Scene-to-ECG intervals by patient characteristics and EMS agency

Variables	Scene-to-ECG interval*, minutes, median [IQR]†	*P* value
Sex		
Male	12.0 [9.0–17.0]	0.001
Female	13.0 [10.0–17.0]	
Age		
< 75 year	12.0 [9.0–17.0]	0.170
* ≥* 75 year	13.0 [9.0–17.0]	
On-scene arrival time‡		
Daytime	12.0 [9.0–16.0]	0.026
Nighttime	13.0 [9.0–18.0]	
EMS agency		
#1	12.0 [9.0–16.0]	< 0.001
#2	13.5 [10.0–19.0]	
#3	17.0 [11.0–28.5]	
#4	10.0 [8.0–14.0]	
#5	15.0 [10.0–22.0]	
#6	15.0 [11.0–21.0]	
#7	13.5 [9.0–22.0]	
#8	16.0 [12.0–22.0]	
#9	10.0 [8.0–15.0]	
#10	16.0 [12.0–21.0]	
#11	13.0 [8.0–21.0]	

* Scene-to-ECG interval: time from EMS on-scene arrival to ECG transmission

† [IQR]: [25th percentile–75th percentile]

‡ On-scene arrival time was categorised by the EMS on-scene arrival timestamp: Daytime, 09:00–17:59; Nighttime, 18:00–08:59 (spanning midnight to the following day)

ECG indicates electrocardiogram; EMS: emergency medical services

### Factors associated with the scene-to-ECG intervals

#### Primary outcome (scene-to-ECG interval ≤ 10 min)

Across Models 1–3, the agency-level cumulative number of ECG transmissions was positively associated with achieving ≤ 10 min (Model 3 adjusted odds ratio, 1.07 per 100 transmissions; 95% confidence interval [CI], 1.02–1.14; Table 4). Female sex, older age, and nighttime arrival were associated with lower odds of achieving the 10-minute target (Table 4).

**Table 4:** Factors associated with achieving a scene-to-ECG Interval ≤ 10 min: logistic mixed-effects models with multiple imputation

	Model 1			Model 2			Model 3		
	OR	95% CI	*P* value	OR	95% CI	*P* value	OR	95% CI	*P* value
Cumulative number of ECG transmissions, by 100 cases	1.07	1.01–1.13	0.022	1.07	1.01–1.13	0.022	1.07	1.02–1.14	0.011
Sex, female	-	-	-	0.66	0.53–0.81	< 0.001	0.66	0.53–0.82	< 0.001
Age, by 10 years	-	-	-	0.92	0.86–0.99	0.020	0.91	0.85–0.97	0.007
On-scene arrival time, nighttime*	-	-	-	-	-	-	0.71	0.58–0.87	0.001

OR indicates Odds ratio

*On-scene arrival time was categorised by the EMS on-scene arrival timestamp: Daytime, 09:00–17:59; Nighttime, 18:00–08:59 (spanning midnight to the following day)

### Secondary outcome (scene-to-ECG interval, minutes)

The cumulative number of ECG transmissions was inversely associated with interval duration in Models 1–3 (e.g., Model 3 β = −0.29 min per 100 transmissions; 95% CI, − 0.39 to − 0.19; Table 5). Older age and nighttime arrival were linked to longer intervals.

**Table 5:** Factors associated with scene-to-ECG interval: linear mixed-effects models with multiple imputation

	Model 1			Model 2			Model 3		
	Parameter estimates	95% CI	*P* value	Parameter estimates	95% CI	*P* value	Parameter estimates	95% CI	*P* value
Cumulative number of ECG transmissions, by 100 cases	−0.27	-0.47 to -0.07	0.010	-0.26	-0.47 to -0.06	0.012	-0.29	-0.39 to -0.19	0.005
Sex, female	-	-	-	0.13	-0.63 to 0.88	0.744	0.09	-0.66 to 0.85	0.811
Age, by 10 years	-	-	-	0.27	0.02 to 0.52	0.034	0.32	0.07 to 0.57	0.013
On-scene arrival time, nighttime*	-	-	-	-	-	-	1.30	0.58 to 2.02	< 0.001

*On-scene arrival time was categorised by the EMS on-scene arrival timestamp: Daytime, 09:00–17:59; Nighttime, 18:00–08:59 (spanning midnight to the following day)

Sensitivity analyses excluding patients with scene-to-ECG intervals ≥ 34 min (97.5th percentile) yielded consistent results (Tables S1 and S2). Complete-case analyses also showed concordant findings (Tables S3 and S4). When on-scene arrival time was alternatively categorised as daytime (07:00–21:59) or nighttime (22:00–06:59), cumulative number of ECG transmissions remained consistently associated with both outcomes, whereas nighttime arrival was not significantly associated with either outcome under this definition (Tables S5 and S6).

## Discussion

In this prefecture-wide cohort of 1748 patients with prehospital ECG transmission, 38.4% (672 of 1748) achieved transmission within 10 min, with a median scene-to-ECG interval of 12 min (IQR 9–17). Greater cumulative EMS-agency experience was associated with shorter scene-to-ECG intervals and higher odds of meeting the 10-minute benchmark. Female sex, older age, and nighttime arrival were linked to lower odds of achieving ≤ 10 min. When modelled as a continuous outcome, older age and nighttime arrival corresponded to longer scene-to-ECG intervals, whereas sex did not. Results were consistent across sensitivity analyses. In descriptive comparisons, patients who achieved the 10-minute benchmark also had shorter on-scene and scene-to-hospital intervals, which may reflect differences in scene complexity, early coordination, transport distance, or receiving hospital availability.

These findings align with prior prehospital studies, in which meeting the ≤ 10-minute target remains uncommon [7, 18, 19, 21]. Although the 10-minute FMC-to-ECG target is guideline-based and provides a useful benchmark for comparison with prior studies, achieving this target in the prehospital setting may be more challenging than in the ED because ECG acquisition and transmission must be performed under variable field conditions. Therefore, the 10-minute threshold may represent an operational benchmark for timely prehospital ECG transmission, rather than a direct equivalent of door-to-ECG performance in the ED.

A related but distinct operational question is whether ECG transmission itself prolongs prehospital care compared with no transmission. A previous retrospective cohort study of patients with ACS suggested that prehospital ECG transmission was associated with a modest increase in scene time of approximately 3 min, although the magnitude of this delay was considered clinically limited [24]. Because all patients in the present analytic cohort underwent completed ECG transmission, the present study could not evaluate this incremental time cost.

Collectively, these results suggest an experience-related association with improved on-scene ECG efficiency. Although variation in case volume across EMS agencies likely reflects differences in catchment populations, ambulance deployment, and local EMS demand, greater cumulative ECG transmissions were associated with shorter scene-to-ECG intervals after accounting for agency-level clustering. Because intermediate workflow elements, such as task delegation, lead placement, and device setup time, were not measured, the mechanisms underlying this association remain uncertain and causal inferences cannot be made. Nevertheless, these findings support structured onboarding, periodic practice, and feedback as pragmatic targets for prospective evaluation, particularly in agencies with less accumulated experience.

Prior ED studies have linked female sex, older age, and atypical presentations to longer door-to-ECG intervals [13, 25–27]. By contrast, standardised triage protocols, staff education, and workflow redesign have been shown to shorten these intervals [14–17]. Evidence regarding the prehospital FMC-to-ECG interval is more limited; however, existing studies similarly report low achievement of the 10-minute benchmark and identify patient-related contributors to delay [7, 18, 19, 21]. Longer intervals among older patients have been documented in previous prehospital studies [18]. This pattern may reflect additional time required for multiple on-scene procedures, including assessment, positioning, intravenous access, and patient transfer [18].

Prior prehospital reports have attributed longer times in females to a higher frequency of atypical symptoms that delay recognition [18, 21]. In our cohort, female sex was associated with lower odds of achieving the 10-minute benchmark, but not with the continuous scene-to-ECG interval. Therefore, this sex-related finding should be interpreted cautiously.

Evidence regarding time-of-day differences in prehospital ECG acquisition remains limited. In one multicentre analysis, the interval from EMS arrival to out-of-hospital ECG was longer during daytime than nighttime [18]. In our main analysis, arrival during the broad nighttime period (18:00–08:59) was associated with longer scene-to-ECG intervals. However, when nighttime was defined more narrowly as 22:00–06:59, nighttime arrival was not significantly associated with scene-to-ECG timing. This suggests that the time-of-day findings should be interpreted cautiously and may reflect broader off-hours conditions, including evening and early-morning periods, rather than late-night hours alone.

This study has several limitations. First, prehospital ECG acquisition was not based on a standardised prefecture-wide protocol, and patient-level symptoms, reasons for ECG acquisition, and clinical severity at the scene were not systematically recorded. Residual selection or indication bias related to ECG acquisition therefore cannot be excluded. In addition, cumulative agency-level ECG transmissions were used as a proxy for organisational experience because individual EMS personnel identifiers, team composition, training history, and education level were unavailable. Therefore, we could not distinguish agency-level experience from individual provider- or team-level effects.

Second, the present study was designed to evaluate prehospital process measures rather than hospital-phase care. Because the registry was not linked to in-hospital records and the transmitted ECGs were not centrally adjudicated, final diagnoses, reperfusion treatment, and clinical outcomes were unavailable. Therefore, the present findings should be interpreted as factors associated with timely ECG transmission, not as evidence that shorter scene-to-ECG intervals led to earlier reperfusion or improved outcomes. Third, this retrospective analysis was conducted within a single Japanese prefecture, which may limit generalisability to systems with different EMS configurations, patient populations, or health care structures. Not all EMS agencies in the prefecture participated (11 of 14), raising the possibility of participation bias and limiting the representativeness of agency-level practices. These findings should therefore be extrapolated cautiously to other settings.

Fourth, because the study period (September 2021–August 2025) overlapped with the COVID-19 pandemic, residual influences of pandemic-related changes in EMS operations and patient characteristics on scene-to-ECG intervals cannot be excluded.

Future studies should use more granular workflow timestamps and linkage with in-hospital data to identify modifiable sources of delay and determine whether improved prehospital ECG timing is associated with reperfusion treatment and clinical outcomes.

## Conclusions

At the EMS agency level, a higher cumulative number of prehospital ECG transmissions was associated with achieving the ≤ 10-minute scene-to-ECG target. This association suggests that focused onboarding and continued EMS training may enhance prehospital ECG performance. In the main analysis, older age, female sex, and nighttime arrival were associated with lower odds of achieving the target.

## Supplementary Information

Below is the link to the electronic supplementary material.

Supplementary Material 1

## Data Availability

The data analysed in this study are derived from the Oita Prehospital ECG Registry. Due to ethical restrictions regarding patient privacy and the terms of approval from the Oita University Human Research Committee, the deidentified individual patient data are not publicly available. Data may be made available from the corresponding author (Hiroki Sato) on reasonable request and following additional approval from the Oita University Human Research Committee.

## Abbreviations

ACS: Acute coronary syndrome
ECG: Electrocardiogram
ED: Emergency department
EMS: Emergency medical services
FMC: First medical contact
PCI: Percutaneous coronary intervention
STEMI: ST-segment elevation myocardial infarction
