# Supplementary material for: Factors associated with achieving the 10-minute scene-to-ECG transmission target in prehospital care: the role of EMS agency-level cumulative ECG transmissions
Source: BMC Emerg Med. 2026 May 29;26:210. doi: 10.1186/s12873-026-01630-8 (PMC13439999; doi:10.1186/s12873-026-01630-8)
Supplement: Supplementary file 1 [file 12873_2026_1630_MOESM1_ESM.pdf]

Table S1. Factors Associated with Achieving a Scene-to-ECG Interval ≤10 Minutes: Logistic Mixed-Effects Models with Multiple Imputation, Excluding ≥97.5th Percentile Cases (≥34 Minutes) (n=1,699)

	Model 1			Model 2			Model 3		
	OR	95% CI	P value	OR	95% CI	P value	OR	95% CI	P value
Cumulative number of ECG transmissions, by 100 cases	1.06	1.01–1.12	0.029	1.06	1.01–1.12	0.032	1.07	1.01–1.13	0.017
Sex, female	-	-	-	0.64	0.52–0.80	<0.001	0.65	0.52–0.80	<.0001
Age, by 10 years	-	-	-	0.93	0.87–0.99	0.033	0.92	0.85–0.98	0.013
On-scene arrival time, nighttime*	-	-	-	-	-	-	0.72	0.58–0.88	0.002

OR indicates Odds ratio.

*On-scene arrival time was categorized by the EMS on-scene arrival timestamp: Daytime, 09:00–17:59; Nighttime, 18:00–08:59 (spanning midnight to the following day).

Table S2. Factors Associated with Scene-to-ECG Interval: Linear Mixed-Effects Models with Multiple Imputation, Excluding ≥97.5th Percentile Cases (≥34 Minutes) (n=1,699)

	Model 1			Model 2			Model 3		
	Parameter estimates	95% CI	P value	Parameter estimates	95% CI	P value	Parameter estimates	95% CI	P value
Cumulative number of ECG transmission, by 100 cases	-0.19	-0.35 to -0.03	0.021	-0.19	-0.35 to -0.03	0.023	-0.21	-0.37 to -0.05	0.012
Sex, female	-	-	-	0.59	-0.01 to 1.19	0.054	0.57	-0.03 to 1.16	0.062
Age, by 10 years	-	-	-	0.12	-0.08 to 0.31	0.248	0.15	-0.05 to 0.35	0.137
On-scene arrival time, nighttime*	-	-	-	-	-	-	0.91	0.34 to 1.48	0.002

*On-scene arrival time was categorized by the EMS on-scene arrival timestamp: Daytime, 09:00–17:59; Nighttime, 18:00–08:59 (spanning midnight to the following day).

Table S3. Factors Associated with Achieving a Scene-to-ECG Interval ≤10 Minutes: Logistic Mixed-Effects Models with Multiple Imputation (Complete Case) (n=1,741)

	Model 1			Model 2			Model 3		
	OR	95% CI	P value	OR	95% CI	P value	OR	95% CI	P value
Cumulative number of ECG transmissions, by 100 cases	1.07	1.01–1.13	0.029	1.07	1.01–1.14	0.028	1.08	1.02–1.15	0.014
Sex, female	-	-	-	0.66	0.53–0.81	0.000	0.66	0.53–0.82	0.000
Age, by 10 years	-	-	-	0.92	0.86–0.99	0.023	0.91	0.85–0.98	0.009
On-scene arrival time, nighttime*	-	-	-	-	-	-	0.71	0.58–0.88	0.001

OR indicates Odds ratio.

*On-scene arrival time was categorized by the EMS on-scene arrival timestamp: Daytime, 09:00–17:59; Nighttime, 18:00–08:59 (spanning midnight to the following day).

Table S4. Factors Associated with Scene-to-ECG Interval: Linear Mixed-Effects Models with Multiple Imputation (Complete Case) (n=1,741)

	Model 1			Model 2			Model 3		
	Parameter estimates	95% CI	P value	Parameter estimates	95% CI	P value	Parameter estimates	95% CI	P value
Cumulative number of ECG transmission, by 100 cases	–0.29	-0.51 to -0.07	0.011	–0.28	-0.50 to -0.06	0.013	–0.31	-0.53 to -0.09	0.006
Sex, female	-	-	-	0.12	-0.63 to 0.88	0.754	0.09	-0.67 to 0.84	0.820
Age, by 10 years	-	-	-	0.26	0.01 to 0.51	0.040	0.31	0.06 to 0.56	0.016
On-scene arrival time, nighttime*	-	-	-	-	-	-	1.27	0.55 to 1.99	0.001

*On-scene arrival time was categorized by the EMS on-scene arrival timestamp: Daytime, 09:00–17:59; Nighttime, 18:00–08:59 (spanning midnight to the following day).

Table S5. Sensitivity Analysis Using Nighttime Defined as 22:00–06:59: Logistic Mixed-Effects Models for Achieving a Scene-to-ECG Interval of ≤10 Minutes (n = 1,699)

	Model 3		
	OR	95% CI	P value
Cumulative number of ECG transmissions, by 100 cases	1.07	1.01–1.13	0.017
Sex, female	0.65	0.53–0.81	<0.001
Age, by 10 years	0.92	0.85–0.98	0.013
On-scene arrival time, nighttime*	0.88	0.63–1.23	0.458

OR indicates Odds ratio.

*On-scene arrival time was categorized by the EMS on-scene arrival timestamp: Daytime, 07:00–21:59; Nighttime, 22:00–06:59 (spanning midnight to the following day).

Table S6. Sensitivity Analysis Using Nighttime Defined as 22:00–06:59: Linear Mixed-Effects Models for Scene-to-ECG Interval (n = 1,699)

	Model 3		
	Parameter estimates	95% CI	P value
Cumulative number of ECG transmission, by 100 cases	-0.27	-0.48 to -0.07	0.009
Sex, female	0.14	-0.61 to 0.90	0.710
Age, by 10 years	0.28	0.03 to 0.53	0.026
On-scene arrival time, nighttime*	0.35	-0.69 to 1.38	0.513

*On-scene arrival time was categorized by the EMS on-scene arrival timestamp: Daytime, 07:00–21:59; Nighttime, 22:00–06:59 (spanning midnight to the following day).
